# *In Vitro* Determination of Genotoxicity Induced by Brackets Alloys in Cultures of Human Gingival Fibroblasts

**DOI:** 10.1155/2020/1467456

**Published:** 2020-08-27

**Authors:** Juan Pablo Loyola-Rodríguez, Ildelfonso Lastra-Corso, José Obed García-Cortés, Alejandra Loyola-Leyva, Rúben Abraham Domínguez-Pérez, David Avila-Arizmendi, Guillermo Contreras-Palma, Cecilia González-Calixto

**Affiliations:** ^1^Universidad Popular Autónoma del Estado de Puebla (UPAEP), Puebla, Mexico; ^2^Universidad Autónoma de San Luis Potosí, San Luis Potosí, Mexico; ^3^CIACYT, Universidad Autónoma de San Luis Potosí, San Luis Potosí, Mexico; ^4^Universidad Autónoma de Quéretaro, Quéretaro, Mexico; ^5^Universidad Autónoma de Guerrero, Acapulco, Guerrero, Mexico

## Abstract

Orthodontic brackets release ions that can be reabsorbed in the oral mucosa, potentially causing complications, including cytotoxic effects and mutagenic alterations. The aim was to evaluate the genotoxicity induced by orthodontic appliance alloys in cultures of human gingival fibroblasts by comet assay. Eluates were obtained from the following brackets alloys: EconoLine (SS: stainless steel), MiniMirage (Ni-Ti: nickel-titanium), Nu-Edge (Co-Cr: cobalt-chromium), In-Vu (PC-polycrystals (PC) aluminum oxide), and Monocrystal IZE (monocrystalline (MC) aluminum oxide). Each bracket was sterilized and exposed to a corrosive process for 35 days. The obtained eluates were tested for genotoxicity of human gingival fibroblasts (HGFA) by the alkaline comet assay. All study groups showed genotoxic effects; there was a significant difference (*p* < 0.0001) among groups. The eluates obtained from Ni-Ti showed a 16-times greater genotoxic effect. There were differences in genotoxicity after comparing the Ni-Ti with SS (*p* < 0.01) and Co-Cr brackets (*p* < 0.001). The ceramic was more genotoxic than metallic brackets (SS and Co-Cr), but less than the Ni-Ti. This *in vitro* model will be useful for further study of early DNA damage caused by brackets and other biomaterials used in the oral cavity before their introduction into the clinical setting.

## 1. Introduction

The appliances used for fixed orthodontic therapy include brackets, bands, and archwires. These are manufactured to have a high corrosion resistance using stainless steel, nickel-titanium, chromium, or nickel-cobalt alloys [1]. Brackets are devices build with adequate designs for each orthodontic technique, and different materials, such as metallic, ceramic, polymeric (polycarbonate and polyurethane), or a combination are used for their fabrication. The alloys of stainless steel (SS) commonly employed contained between 15 and 54% of nickel (Ni), 20–30% of chromium (Cr), and 40–60% of cobalt (Co). Ceramic brackets present optical and mechanical characteristics superior to polymeric brackets, and they can be classified according to their fabrication into polycrystalline aluminum oxide (Al_2_O_3_P) and monocrystalline aluminum oxide (Al_2_O_3_M) [2, 3].

The use of orthodontic appliances results in the release of metallic ions, such as nickel, chromium, and cobalt, into the oral cavity, which is of significant clinical concern. Electrochemical corrosion could occur when metals are in contact with mediums of electrolytic conductivity, especially in saliva and oral tissues. In this situation, metals are exposed to a degradation process promoted by thermal, microbiological, and enzymatic properties due to chemical reactions caused by food and beverage consumption [2]. Due to corrosion, metal alloys employed in orthodontics could cause an indirect effect on the DNA of cells by generating free radicals with mutagenic or carcinogenic effects. These results are due to the entry, accumulation, and the absorption of free radicals into oral and organism tissues (gingival, oral, intestinal, cutaneous, and respiratory system epithelium) [2, 4].

Some components of metallic alloys, especially Ni, Cr, and Co, can cause disturbances, such as hypersensibility, immunological sensibility, dermatitis, hyperplasia, gingivitis, and even asthma. Cobalt is an element capable of inducing abnormal mitosis, and Ni can inhibit cellular proliferation, lactate production, and consumption of glucose [2, 5]. Both metals are classified as carcinogenic because, *in vitro*, they are capable of causing harmful effects in human gingival mast cells and fibroblasts affecting their proliferation, morphology, and production of collagen [6, 7].

Cytotoxicity is a type of damage to the cell membrane exposing its cytoplasm causing cell death. Furthermore, genotoxicity is direct or indirect damage to cellular genetic material, which represents an essential part of assessing oral cancer risk and carcinogenic potential. In the oral cavity, these types of damage are caused by an increase in free radical production, mainly nickel, steel, and chromium ions. In the manufacture of brackets, different metallic materials and ceramic, polymeric, or combinations of materials are used. Due to this variety, the present study assessed various alloys of devices, such as metallic and ceramic, avoiding the use of recycled brackets [7]. A wide range of methods is used to detect early biological effects of DNA-damaging agents in environmental and occupational settings. There are well-established cytogenetic biomarkers, such as structural chromosomal aberrations, micronuclei, sister chromatid exchanges, and high-frequency cells [8]. However, during the last decade, the comet assay (CA or single cell gel electrophoresis) was based on the principle that damaged DNA moves faster than undamaged DNA in an agarose gel. A cell with DNA damage appears in the form of a comet, whereas an intact DNA appears as a halo [9]. An advantage over the cytogenetic biomonitoring techniques (micronuclei test: MNs) is that MNs are limited to circulating and proliferating cell populations, whereas the CA can be applied to both proliferating and nonproliferating cells.

There are reports that have addressed the genotoxicity induced by orthodontic materials, mainly analyzed by MNs [10, 11], but few studies have addressed this using CA [12]. An important point to note is that it is necessary to establish a rapid *in vitro* assay to test the biocompatibility of the different biomaterials used in clinical orthodontics. Therefore, this study aimed to analyze and compare the genotoxicity induced by eluates obtained from various brackets alloys using human gingival fibroblasts by the comet assay test.

## 2. Materials and Methods

### 2.1. Primary Culture of Human Gingival Fibroblasts Assay (HGFA)

Gingival biopsies were harvested from healthy donors who underwent a crown lengthening surgery for prosthetic reasons. Informed consent and research protocol were institutionally approved. The tissue sample was placed in 1 mL of DMEM (Dulbecco's Modified Eagle Medium, Corning Life Sciences, NY, USA) supplemented with 10% fetal bovine serum (FBS) and antibiotics (penicillin 100 IU/ml, streptomycin 100 *μ*g/ml, and amphotericin B 100 *μ*g/ml). Collected tissues were washed twice with phosphate-buffered saline (PBS; 150 mM NaCl, 20 mM sodium phosphate, pH 7.2) supplemented with antibiotics (penicillin 100 IU/ml and streptomycin 100 *μ*g/ml) and, then, cut into small pieces with a sterile surgical blade in a Petri dish. Tissue fragments were further digested by collagenase/dispase (Hoffmann-La Roche Corporation, Tucson, AZ, USA) for 1 h at 37°C in 100% of humidity, 95% oxygen, and 5% CO_2_. Minced pieces of gingival tissue were washed with DMEM and FBS (10%) and explanted to 25 cm^2^ tissue culture flasks (Falcon, Corning Life Sciences, NY, USA) containing 5 ml of supplemented DMEM and incubated at 37°C in 100% of humidity, 95% oxygen, and 5% CO_2_. HGF was obtained by trypsinization of the primary cell outgrown and routinely passaged using 0.025% trypsin in PBS containing 0.02% EDTA at 90% of cell confluence. HGF between the 6^th^ and 8^th^ passages were used in the experiments. The cellular count was made using a Neubauer camera, and 1 × 10^4^ cells per well were cultured in plates of 48 wells (Falcon, Corning Life Sciences, NY, USA) [13].

### 2.2. Sample Preparation

The brackets were immersed in a solution containing acetic acid (0.1 M) and sodium chloride (0.1 M) to produce a corrosion process, and each sample was carried out by triplicate. Samples were maintained for 35 days at room temperature and, then, sterilized with ultraviolet light. HGF cultures were exposed to 200 *μ*l of eluates for 1 h at 37°C. After exposure, HGF was washed twice with phosphate buffered solution (PBS) [14]. Cells were treated with the same solution used for corrosion (without brackets) for the positive control group, and saline was used as the negative control.  Table 1 shows the properties of the brackets analyzed in this study.

### 2.3. Genotoxicity Assay

To evaluate the magnitude of DNA damage, the alkaline version of a single cell gel (comet) assay was used. To break the cell membrane, HGF was fixed in a low melting point agarose and exposed to a cell lysis solution for 24 h. Next, the cells were submitted to an unwinding solution for 20 min to denature the DNA and free the broken fragments that suffered damage. Finally, exposed cells were subjected to electrophoresis for 20 min at 25 V and 300 mA, in which the particles migrate towards the anode, making a tail, giving the cells an appearance of a comet. To determine DNA damage, samples were stained with ethidium bromide and visualized in a fluorescence microscope. A total of 100 cells per sample were analyzed (*n* = 10 per group). The damage was analyzed using the software Comet Assay IV (Perceptive Instruments Ltd., Haverhill, UK); this software allows the capture and analysis of images from comet assay that permits the quantification of DNA damage and repair in a single cell preparation. Tail moment was calculated by the image analysis system as the product of the tail length (DNA migration) and the fraction of DNA in the comet tail (% DNA in the tail).

### 2.4. Statistical Analysis

Statistical software GraphPad InSat 3.1 (GraphPad Software, Inc.) was used to express data as means and standard deviations. The Kolmogorov–Smirnov test was used to determine data distribution. The Kruskal–Wallis test followed by Dunn post hoc analysis was used to assess the difference between groups. A *p* value lower than 0.05 was considered statistically significant.

## 3. Results

### 3.1. Genotoxicity Assay

**Figure 1:**
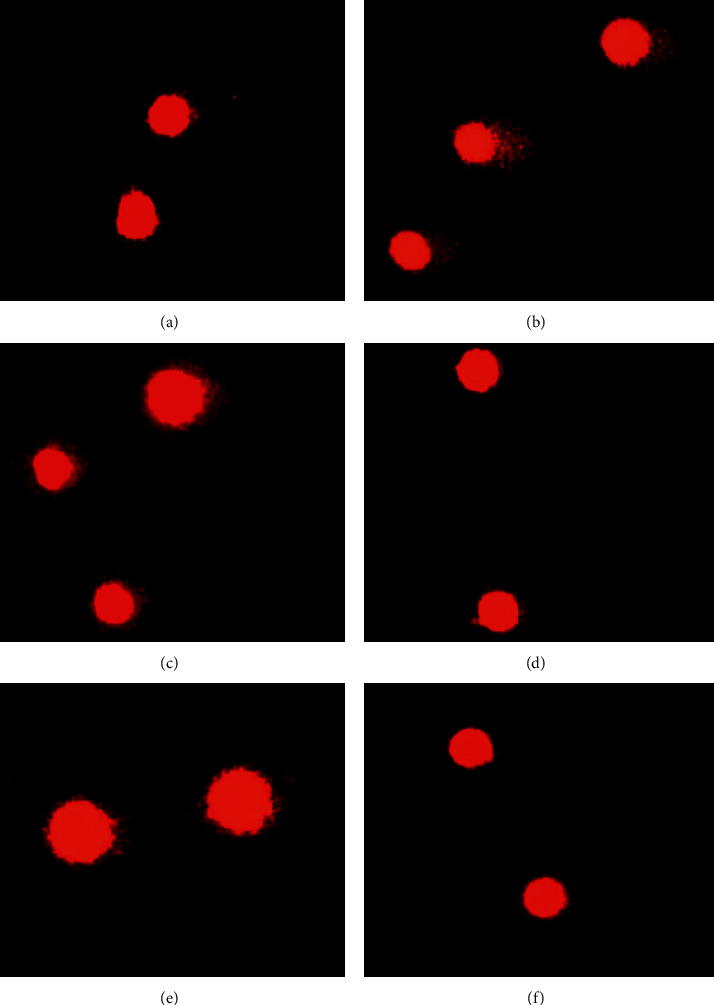
Representative pictures of the damage (genotoxicity effect on HGFA) caused by the exposure to different eluates from brackets as follows: SS (a), Ni-Ti showed the highest DNA damage (b), Co-Cr showed the lowest DNA damage (c), PC-AO (d), MC-AO (e), and control (f).

Table 2 shows the DNA damage in HGFA in the study groups compared with the control; all groups showed biological effects evaluated by alkaline comet assay. However, the Co-Cr group showed less fibroblast DNA damage (0.724 ± 0.015). Besides, the eluates obtained from the analyzed brackets induced up to 3 times more damage. The greatest damage was detected in the samples exposed to the Ni-Ti solution (3.588 ± 0.137), which was approximately 17 times more genotoxic than the control (Table 2 and Figure 1).

Table 3 shows a comparison of the genotoxicity between the different brackets alloys studied. We detected significant differences in the Ni-Ti brackets compared with SS (*p* < 0.01), Co-Cr (*p* < 0.001), and with the control group. Likewise, we detected a significant difference between Co-Cr and MC-AO groups (*p* < 0.01). The comparison of MC-AO and PC-AO groups showed a statistically significant difference (*p* < 0.001) when compared with the control group. However, there was no statistical difference between MC-AO and PC-AO groups (*p* > 0.05). Therefore, it is assumed that ceramic brackets (MC-AO, PC-AO) are most genotoxic than the metallic brackets (SS and Co-Cr), but less than the Ni-Ti.

## 4. Discussion

Ulcers and periodontal pathologies are the main clinical concerns regarding the effects of corrosion of the orthodontic devices due to damage caused to the tissues of the oral cavity [8, 15]. Therefore, it is essential to determine the levels of biocompatibility because brackets are placed in the proximity of the gum and even within the gum (when retained dental organs are pulled). Any metal placed within the oral cavity will suffer corrosion due to the formation of organometallic compounds, which depends on composition, temperature, humidity, and pH [16]. Furthermore, there are intrinsic (material composition, surface roughness, hardness, and other orthodontic devices) and extrinsic factors (cell renewal, proteins, enzymes, ions in saliva, bacteria, friction between orthodontic devices, toothpaste, and some mouthwashes) that contribute to the corrosion process [17, 18].

*In vitro* [19] and *in vivo* [20, 21] studies have evaluated the release of metals such as iron, nickel, and chrome from permanent orthodontic devices. It has been reported that ion release occurs during the first months of orthodontic treatment producing cytotoxic, immunogenic, and mutagenic effects. These effects are caused because materials accumulate in the adjacent soft tissue and could be absorbed, especially Ni-Ti ions through the transport system Mg_2_^+^, calcium, and iron channels or by phagocytosis. Furthermore, it has been reported that Ni-Ti induces apoptosis (by the inhibition of Ni_3_^+^) among gingival fibroblasts, and that this involves caspase-3 activation [22].

There are reports of cellular models about the biocompatibility of different types of metallic and ceramic brackets [23, 24]. However, these studies only assess macrostructure and cell functions (viability and morphology), but not genotoxicity. Conversely, it has also been noted that some brackets are cytotoxic and genotoxic for fibroblast cultures, except titanium brackets [25]. Considering that brackets from different manufacturers have different corrosion behavior, it has been reported that Fe and Cr ions had the most abundant corrosion products of the evaluated materials [17, 19, 23]. However, the brackets that had more significant damage to their external structure were, in ranked order, NiTi, Co-Cr, SS, MC-AO, and PC-AO (data not shown).

There is controversy regarding the evaluation of genotoxicity in oral epithelial cells [2, 26]. Mouse lymphoma L5178Y or Chinese Hamster Ovary A common cells are commonly used for *in vitro* studies due to their ease of handling and cultivation [14]. However, human gingival fibroblasts are more sensible to evaluate side effects of dental materials compared to cell lines derived from animal tissues [27]. An important point to note is that HGFA could be useful to analyze the genotoxic effects of different metal appliances (implants, orthodontic appliances, and oral surgery appliances) that generate nanoparticles and micron-sized particles released in oral tissues [27].

A few studies have assessed the *in vitro* genotoxicity of SS brackets and other metal alloys and ceramics; there is information about the cytotoxicity of polycarbonate and ceramic brackets, but not about genotoxicity. In agreement with the present results, it has been reported that metal brackets produced genotoxicity in HGF when the comet assay was used [25] and others have observed that nanoparticles of cobalt-chromium induced increased DNA damage in cell cultures [35]. By contrast, it has been shown that none of these cited alloys cause DNA alterations in immortalized gingival keratinocytes [17]. The comet assay is a sensitive method to determine the genotoxicity throughout the detection of a primary lesion in the DNA chain of individual cells, which repair efficiently 1 h after their exposure to a toxic agent, mainly when it occurs in regions of active transcription. Primary lesions and double-strand breaks represent initial DNA damage events that can lead to chromosomal aberrations [12, 14, 28]. It has been described that Olive Tail Moment is one of the best indicators of DNA damage which is a practical measure to calculated DNA migration distance as a genotoxic effect [12].

Alloys with a high content of Ni are more susceptible to corrosion than steel, chromium, and magnesium alloys [15, 17, 25]. It has been reported that Ni is the most cytotoxic, and it releases ions that penetrate cells and subsequently affect the functionality and causes hypersensitivity reactions, such as dermatitis, stomatitis, asthma, and burning sensation in the esophagus and neck areas. Besides, the Ni cytotoxic effect reduces the sense of taste and acts as carcinogenic in the nasal cavity and respiratory system [25]. Manganese is a corrosion product from SS brackets that leads to mitochondrial dysfunction that could result in cellular oxidative stress. Producing intra- and extracellular accumulation of glycosaminoglycans (GAG) affects adhesion, migration, growth, and cellular differentiation [23, 29]. Therefore, other alloys, such as pure titanium (that is more inert and stable in the oral environment than SS), have been introduced. Titanium has high corrosion resistance, improved biocompatibility, and does not produce allergic responses [15].

On the other hand, ceramic brackets made of aluminum oxide can cause numerical chromosomal aberrations, and they are not inert materials, as previously thought. However, it has been reported that such brackets show higher biocompatibility in cell cultures due to the monocrystalline composition, which eliminates contamination or impurities during the manufacture. All brackets are a source of oxidative stress; conventional ceramic brackets are more biocompatible compared to polymeric and metal brackets [29].

The comet assay is a rapid, simple, and sensitive technique for measuring DNA breakage with a small number of cells and detects intercellular differences in DNA damage [30]. *In vitro* studies are simple and inexpensive, provide a significant amount of information, and are suited to elucidating the mechanisms of cellular toxicity presented *in vivo* [14]. Therefore, we believe that further *in vitro* studies must be performed with different brands of brackets to determine the type of production and quality of the corrosion resistance. Also, it will be necessary to detect and quantify the ions present and observe the type of genotoxic damage in oral tissues.

## 5. Conclusions

All types of orthodontic brackets, regardless of the constituent materials, produce DNA damage when its eluates are exposed to HGFA using the comet assay test. These findings show that ceramic brackets were more genotoxic than metallic brackets but less than the Ni-Ti. This *in vitro* model will be useful for further study of early DNA damage caused by brackets and other biomaterials used in the oral cavity before their introduction into the clinical setting.

## Figures and Tables

**Table 1:** Type and composition of brackets used in the study.

Brackets	Composition	Made by
EconoLine 0.022	Stainless steel (SS)	Ah-Kim-Pech, Miami, FL, USA
MiniMirage 0.022	Nickel titanium (Ni-Ti)	Borgatta specialties, Estado de México, México
Nu-Edge 0.022	Cobalt-chromium (Co-Cr)	TP orthodontics, Inc., La Porte, IN, USA
In-Vu 0.022	Polycrystalline aluminium oxide (PC-AO)	TP orthodontics, Inc., La Porte, IN, USA
Crystall IZE 0.022	Monocrystalline aluminium oxide (MC-AO)	Ah-Kim-Pech, Miami, FL, USA

**Table 2:** Genotoxicity (comet assay: tail moment) in cultures of human gingival fibroblasts induced by eluates obtained from brackets.

Bracket alloys	Comet assay	Increment of genotoxicity	^*∗*^ *p* value
SS	0.805 ± 0.002	3.79	<0.0001
Ni-Ti	3.588 ± 0.137	16.92	
Co-Cr	0.724 ± 0.015	3.41	
PC-AO	0.828 ± 0.019	3.90	
MC-AO	0.832 ± 0.012	3.92	
Positive control	0.212 ± 0.009		

Kruskal–Wallis. SS: stainless steel; Ni-Ti: nickel-titanium; Co-Cr; cobalt chromium; PC-AO: polycrystalline Al_2_O_3_; MC-AO: monocrystalline Al_2_O_3_; comet assay: DNA damage percentage is expressed in mean and standard deviation. *n* = 10 per each group.

**Table 3:** Comparisons of the difference in genotoxicity among the different brackets alloys used.

Brackets alloys	Difference	*p* value
Stainless steel vs. Ni-Ti	2.783	<0.01
Stainless steel vs. Co-Cr	0.081	>0.05
Stainless steel vs. PC-Al_2_O_3_	0.023	>0.05
Stainless steel vs. MC-Al_2_O_3_	0.027	>0.05
Stainless steel vs. control	0.593	>0.05
Ni-Ti vs. Co-Cr	2.864	<0.001
Ni-Ti vs. PC-Al_2_O_3_	2.76	>0.05
Ni-Ti vs. MC-Al_2_O_3_	2.756	>0.05
Ni-Ti vs. control	3.376	<0.001
Co-Cr vs. PC-Al_2_O_3_	0.104	>0.05
Co-Cr vs. MC-Al_2_O_3_	0.108	<0.01
Co-Cr vs. control	0.512	>0.05
PC-Al_2_O_3_ vs. MC-Al_2_O_3_	0.004	>0.05
PC-Al_2_O_3_ vs. control	0.616	<0.001
MN-Al_2_O_3_ vs. control	0.620	<0.001

Dunn's test. Ni-Ti: nickel-titanium; Co-Cr; cobalt chromium; PC-Al_2_O_3_: polycrystalline aluminium oxide; MC-Al_2_O_3_: monocrystalline aluminium oxide.

## Data Availability

The comet assay data used to support the findings of this study are available from the corresponding author upon request.
