# Corrigendum to “*In Vitro* Determination of Genotoxicity Induced by Brackets Alloys in Cultures of Human Gingival Fibroblasts”

**DOI:** 10.1155/2021/4028481

**Published:** 2021-01-04

**Authors:** Juan Pablo Loyola-Rodríguez, Ildelfonso Lastra-Corso, José Obed García-Cortés, Alejandra Loyola-Leyva, Rúben Abraham Domínguez-Pérez, David Avila-Arizmendi, Guillermo Contreras-Palma, Cecilia González-Calixto, Gabriela Cortés Sandoval

**Affiliations:** ^1^Universidad Popular Autónoma del Estado de Puebla (UPAEP), Puebla, Mexico; ^2^Universidad Autónoma de San Luis Potosí, San Luis Potosí, Mexico; ^3^CIACYT, Universidad Autónoma de San Luis Potosí, San Luis Potosí, Mexico; ^4^Universidad Autónoma de Quéretaro, Quéretaro, Mexico; ^5^Universidad Autónoma de Guerrero, Acapulco, Guerrero, Mexico; ^6^Doctorado Institucional en Ingeniería y Ciencia de Materiales, Universidad Autónoma de San Luis Potosí, Av. Dr. Manuel Nava # 6, Zona Universitaria, CP 78290, San Luis Potosi, SLP, Mexico

In the article titled “*In Vitro* Determination of Genotoxicity Induced by Brackets Alloys in Cultures of Human Gingival Fibroblasts” [1], Dr. Gabriela Cortés Sandoval was missing from the author list. Dr. Gabriela Cortés Sandoval contributed to the experimental design, data collection, and analysis, and the corrected author list is shown above.
